# Increased thyroid hormone sensitivity is correlated with visceral obesity in patients with type 2 diabetes

**DOI:** 10.1186/s12944-024-02320-9

**Published:** 2024-10-16

**Authors:** Lu Yu, Yujia Liu, Yingxuan Wang, Gang Wang, Xianchao Xiao, Huan Wang, Hanyu Wang, Hui Sun, Guixia Wang

**Affiliations:** 1 Department of Endocrinology and Metabolism, The First Hospital of Jilin University, Changchun, 130021 China; 2 Department of Endocrinology and Metabolism, Union Hospital, Tongji Medical College, Huazhong University of Science and Technology, Wuhan, 430000 China

**Keywords:** Sensitivity to thyroid hormones, Visceral fat area, Visceral obesity, Thyroid feedback quantile-based index, Free triiodothyronine to free thyroxine

## Abstract

**Objective:**

The study aimed to assess whether thyroid hormone (TH) sensitivity is related to visceral fat area (VFA) and visceral obesity in euthyroid subjects with type 2 diabetes (T2D).

**Methods:**

750 euthyroid patients with T2D were enrolled. A VFA of 80 cm^2^ or more was considered visceral obesity. Central TH sensitivity was conducted using thyrotrophic thyroxine resistance index (TT4RI), thyrotropin index (TSHI), and thyroid feedback quantile-based index (TFQI). Free triiodothyronine to free thyroxine (FT3/FT4) was utilized for assessing peripheral TH sensitivity.

**Results:**

The subjects had a mean age of 51.5 ± 11.1 years, and 540 (72.0%) of them were men. In multivariable regression analyses, there was a positive correlation of FT3/FT4 tertile with visceral obesity, after full adjustment for confounding variables (*P* < 0.05). The middle and highest FT3/FT4 tertiles were correlated with a 134% [95% CI (1.24, 4.44)] and 98% [95% CI (1.04, 3.78)] higher prevalence of visceral obesity than the lowest tertile, respectively. Conversely, elevated TFQI levels were linked to a decreased prevalence of visceral obesity. Stratified analysis revealed that these associations were particularly pronounced in participants who are neither overweight nor obese and those aged less than 60 years (all *P* < 0.05).

**Conclusions:**

Higher TH sensitivity is correlated with visceral obesity and elevated VFA in euthyroid patients with T2D, particularly among those younger than 60 years and individuals who are neither overweight nor obese.

**Supplementary Information:**

The online version contains supplementary material available at 10.1186/s12944-024-02320-9.

## Introduction

The overweight and obesity pandemics affect more and more population. Even more disturbing is the surge in global obesity rates among children and adolescents [1]. Visceral obesity is a key risk factor for obesity-related ailments, including cardiovascular disorders, type 2 diabetes (T2D), and multiple cancers [2, 3]. In patients with T2D, increased visceral fat accumulation is linked to a greater risk of cardiometabolic disorders [4, 5]. This highlights the urgency for further research into the determinants of visceral obesity to better combat the growing obesity epidemic worldwide.

Thyroid hormones (THs) are crucial for regulating metabolism, energy homeostasis, and cardiovascular health [6]. They are known to enhance metabolic rates, boost energy expenditure, stimulate adaptive thermogenesis in brown adipose tissue and enhance the conversion of white adipose tissue into a more metabolically active state [6–8]. Previous studies have linked hypothyroidism with dyslipidemia and obesity [9]. While hyperthyroidism is correlated with decreased visceral fat deposits [10]. However, the effects of THs can vary significantly across different tissues due to variations in deiodinase levels, TH transporter activity, TH receptor isoforms, and the overall number of TH receptors among other factors [11–14]. This variability underscores the limitations of using serum TH levels alone to accurately assess thyroid status in specific tissues like adipose tissue. Therefore, evaluating TH sensitivity is crucial for a thorough assessment of both systemic and tissue-specific thyroid function. For this purpose, indices such as the thyrotrophic thyroxine resistance index (TT4RI), thyrotropin index (TSHI), and thyroid feedback quantile-based index (TFQI) are commonly employed to gauge central TH sensitivity, while free triiodothyronine to free thyroxine (FT3/FT4) is utilized for assessing peripheral TH sensitivity [15–17].

Several studies have indicated that impaired central TH sensitivity is linked to greater risk of metabolic syndrome, diabetes [15], hyperuricemia [18], cardiovascular disease [19, 20], and elevated visceral fat area (VFA) [21]. However, the correlation between VFA and FT3/FT4 remains controversial, with findings ranging from positive [22], to uncorrelated [23], to negative [21]. More importantly, TH and adipose tissue have a profound effect on energy metabolism in patients with diabetes [3, 24]. Whether TH sensitivity correlates with VFA and visceral obesity in patients with T2D remains unexplored. We hypothesize that TH sensitivity varies dynamically across different disease states, especially in metabolic abnormalities such as diabetes or obesity.

Therefore, this research focused on the connection between TH sensitivity with VFA and visceral obesity in euthyroid individuals with T2D, thereby identifying potential therapeutic targets for diabetic patients with visceral obesity to enhance metabolic health by regulating energy homeostasis.

## Methods

### Participants

From May 2017 to April 2019, 1,136 adult inpatients with T2D were enrolled from the National Metabolic Management Center [25] at the First Bethune Hospital of Jilin University. Individuals were excluded for the reasons below: acute complications of diabetes (*n* = 20), a self-reported thyroid disease, including subclinical thyroid dysfunction (*n* = 37), undergoing anti-thyroid therapy or TH replacement therapy (*n* = 23), missing data on thyroid function (*n* = 91) or VFA (*n* = 97), or having FT3, thyrotropin (TSH), or FT4 levels beyond normal reference ranges (*n* = 118). Ultimately, the study comprised 750 participants, all of whom provided written informed consent.

## Data collection

A standardized questionnaire was administered one-on-one by trained staff to collect information, including demographic data, disease history, and medication history. Body mass index (BMI) was calculated as weight (kg)/height (m)^2^. Overweight was classified as 25 kg/m^2^ ≤ BMI < 30 kg/m^2^ and obesity as BMI ≥ 30 kg/m^2^ [26]. The normal reference ranges, quantified by supersensitive electrochemiluminescence immunoassay (Siemens Centaur XP, Germany), were 3.1–6.8 pmol/L for FT3, 0.27–4.2 mIU/L for TSH, and 12–22 pmol/L for FT4. Bioelectrical impedance analyses (DUALSCAN HDS-2000) was utilized to assess VFA and subcutaneous fat area (SFA) [27].

Any of the criteria below were used to define dyslipidemia: high-density lipoprotein cholesterol (HDL-C) < 1.04 mmol/L, low-density lipoprotein cholesterol (LDL-C) ≥ 3.4 mmol/L, triglycerides (TG) ≥ 1.7 mmol/L, total cholesterol (TC) ≥ 5.2 mmol/L, or using lipid-lowering agents [28]. Any of the criteria below were used to define hypertension: using antihypertensive agents, having a previous diagnosis of hypertension, diastolic blood pressure (DBP) ≥ 90 mmHg, or systolic blood pressure (SBP) ≥ 140 mmHg [29]. A VFA of 80 cm^2^ or more was considered visceral obesity [30, 31].

## Definition of TH sensitivity

TSHI was calculated as Ln TSH (mIU/L) + 0.1345 × FT4 (pmol/L) [17]. TFQI was calculated as empirical cumulative distribution function (cdf) FT4 − (1 − cdf TSH) [15]. TT4RI was calculated as FT4 (pmol/L) × TSH (mIU/L) [16]. Higher positive values of TFQI, TSHI and TT4RI indicate a greater impairment in central TH sensitivity. FT3/FT4 is utilized for assessing peripheral TH sensitivity.

### Statistical analysis

The correlations of TSHI, TT4RI, TFQI, FT3/FT4 with both VFA and visceral obesity were examined using multivariable regression models. Age and sex were adjusted in model 1. Further adjustments in model 2 included BMI, glycated hemoglobin A1c (HbA1c), uric acid, duration of diabetes, fasting plasma glucose (FPG), dyslipidemia, hypertension, use of lipid-lowering agents, antihypertensive agents, glucagon-like peptide-1 receptor agonist (GLP-1 RA) therapy, metformin therapy, drinking and smoking status. Stratified analyses were conducted by BMI groups (< 25 kg/m^2^, ≥ 25 kg/m^2^), sex and age categories (< 60 years, ≥ 60 years). In order to investigate the possible nonlinear relationship, smoothing and generalized additive model was used.

Statistical analyses were performed using R package (http://www.Rproject. org) and Empower Stats (http://www.empowerstats.com). *P* < 0.05 was deemed statistically significant.

## Results

### Baseline characteristics

The baseline characteristics of subjects are outlined in Table 1, categorized by VFA < 80 cm^2^ and VFA ≥ 80 cm^2^. The study subjects had a mean age of 51.5 ± 11.1 years and a mean diabetes duration of 98.45 ± 86.31 months, 540 (72.0%) of whom were men. Compared to those with VFA < 80 cm^2^, the participants in the group with VFA ≥ 80 cm^2^ had a higher prevalence of male gender, smoking, and alcohol consumption; more frequently used antihypertensive and lipid lowering agents, metformin and GLP-1 RA; and had higher DBP, SBP, BMI, waist circumference (WC), FT3/FT4, FT3, uric acid, and SFA levels (all *P* < 0.05). However, between the two groups, TFQI, TT4RI, or TSHI did not differ significantly.

**Table 1:** Basic participant characteristics categorized by VFA

	Overall	VFA < 80 cm^2^	VFA ≥ 80 cm^2^	*P* value
N	750	168	582	
Age (year)	51.54 ± 11.07	51.56 ± 10.96	51.54 ± 11.11	0.913
Male, n (%)	540 (72.00)	99 (58.93)	441 (75.77)	< 0.001
Duration of diabetes (month)	98.45 ± 86.31	96.85 ± 82.48	98.92 ± 87.46	0.954
DBP (mmHg)	78.18 ± 10.94	74.89 ± 11.04	79.13 ± 10.74	< 0.001
SBP (mmHg)	129.42 ± 16.99	125.13 ± 18.75	130.66 ± 16.25	< 0.001
BMI (kg/m^2^)	27.30 ± 14.08	23.69 ± 3.07	28.34 ± 15.75	< 0.001
Waist circumference (cm)	95.87 ± 9.21	86.68 ± 7.00	98.54 ± 7.98	< 0.001
FPG (mmol/ L)	8.54 ± 3.23	8.16 ± 3.14	8.65 ± 3.25	0.062
HbA1C (%)	8.93 ± 2.10	8.88 ± 2.28	8.94 ± 2.05	0.484
Uric acid (umol/ L)	337.39 ± 86.21	294.48 ± 80.05	349.46 ± 84.07	< 0.001
Triglyceride (mmol/l)	1.86 (1.30, 2.91)	1.35 (1.02, 1.96)	2.02 (1.43, 3.17)	< 0.001
HDL cholesterol (mmol/ L)	1.12 ± 0.29	1.26 ± 0.33	1.08 ± 0.26	< 0.001
LDL cholesterol (mmol/ L)	2.84 ± 0.83	2.87 ± 0.86	2.83 ± 0.83	0.607
Total cholesterol (mmol/ L)	4.78 ± 1.19	4.79 ± 1.15	4.78 ± 1.21	0.856
FT3 (pmol/L)	4.61 ± 0.62	4.45 ± 0.62	4.65 ± 0.61	< 0.001
FT4 (pmol/L)	16.19 ± 2.26	16.36 ± 2.31	16.15 ± 2.25	0.317
TSH (mIU/L)	2.02 (1.39, 2.71)	2.02 (1.34, 2.65)	2.02 (1.40, 2.73)	0.399
FT3/FT4	0.29 ± 0.05	0.28 ± 0.05	0.29 ± 0.04	< 0.001
TFQI	0.42 (0.18, 0.69)	0.48 (0.16, 0.69)	0.41 (0.19, 0.70)	0.962
TT4RI	31.75 (22.08, 43.60)	30.82 (21.29, 43.79)	31.78 (22.32, 43.54)	0.605
TSHI	2.85 (2.47, 3.20)	2.84 (2.40, 3.23)	2.85 (2.49, 3.19)	0.974
VFA (cm^2^)	109.47 ± 39.28	59.86 ± 14.59	123.79 ± 31.79	< 0.001
SFA (cm^2^)	196.76 ± 62.32	141.77 ± 41.99	212.53 ± 58.12	< 0.001
Smoking, n (%)	240 (32.17)	40 (23.95)	200 (34.54)	0.010
Drinking, n (%)	157 (21.07)	19 (11.45)	136 (23.49)	< 0.001
Hypertension, n (%)	282 (37.60)	47 (27.98)	235 (40.38)	0.003
Dyslipidemia, n (%)	318 (42.40)	51 (30.36)	267 (45.88)	< 0.001
Antihypertensive agents, n (%)	255 (34.00)	43 (25.60)	212 (36.43)	0.009
Lipid lowering agents, n (%)	197 (26.30)	30 (17.86)	167 (28.74)	0.005
Metformin therapy, n (%)	237 (35.27)	34 (22.52)	203 (38.96)	< 0.001
GLP-1 RA therapy, n (%)	9 (1.34)	0 (0.00)	9 (1.34)	0.047

Data are presented as mean ± standard deviation (SD), median (interquartile range), or n (%)

*Abbreviations*: VFA, visceral fat area; DBP, diastolic blood pressure; SBP, systolic blood pressure; BMI, body mass index; FPG, fasting plasma glucose; HbA1c, glycated hemoglobin; HDL, high-density lipoprotein; LDL, low-density lipoprotein; FT3, free triiodothyronine; FT4, free thyroxine; TSH, thyrotropin; TFQI, thyroid feedback quantile-based index; TT4RI, thyrotrophic thyroxine resistance index; TSHI, TSH index; SFA, subcutaneous fat area; GLP-1 RA, glucagon-like peptide-1 receptor agonist

Table 2 presents the characteristics of subjects divided into tertiles of FT3/FT4. The highest FT3/FT4 tertile exhibited a larger proportion of smokers and drinkers; exhibited higher DBP, SBP, WC, uric acid, VFA, and SFA levels, but lower levels of HDL-C, FPG, HbA1c, and TC than the lowest tertile (all *P* < 0.05).

**Table 2:** Participant characteristics classified by FT3/FT4 tertiles

	T 1	T 2	T 3	*P* value
	0.17–0.27	0.27–0.31	0.31–0.43	
N	250	250	250	
Age (year)	50.90 ± 11.76	52.50 ± 10.89	51.22 ± 10.51	0.233
Male, n (%)	156 (62.40)	184 (73.60)	200 (80.00)	< 0.001
Duration of diabetes (month)	94.70 ± 90.70	98.53 ± 82.96	102.19 ± 85.32	0.642
DBP (mmHg)	76.57 ± 10.44	78.89 ± 11.98	79.08 ± 10.17	0.017
SBP (mmHg)	126.99 ± 16.86	130.34 ± 17.07	130.94 ± 16.84	0.020
BMI (kg/m^2^)	25.88 ± 3.27	28.16 ± 21.40	27.85 ± 11.13	0.146
Waist circumference (cm)	93.69 ± 9.64	97.06 ± 9.24	96.89 ± 8.33	< 0.001
FPG (mmol/ L)	9.12 ± 3.79	8.33 ± 2.67	8.17 ± 3.06	0.002
HbA1C (%)	9.62 ± 2.37	8.74 ± 1.95	8.42 ± 1.76	< 0.001
Uric acid (umol/ L)	324.87 ± 86.88	346.54 ± 87.52	340.55 ± 83.00	0.020
Triglyceride (mmol/l)	1.78 (1.21, 2.48)	1.95 (1.35, 3.22)	1.91 (1.38, 2.92)	0.022
HDL cholesterol (mmol/ L)	1.16 ± 0.30	1.11 ± 0.32	1.09 ± 0.24	0.024
LDL cholesterol (mmol/ L)	2.95 ± 0.87	2.77 ± 0.79	2.80 ± 0.84	0.052
Total cholesterol (mmol/ L)	4.94 ± 1.31	4.73 ± 1.15	4.68 ± 1.11	0.048
FT4 (pmol/L)	17.59 ± 2.15	16.34 ± 1.99	14.65 ± 1.58	< 0.001
TSH (mIU/L)	1.90 (1.24, 2.57)	2.05 (1.40, 2.70)	2.04 (1.51, 2.79)	0.045
VFA (cm^2^)	98.94 ± 38.59	114.71 ± 39.88	114.75 ± 37.33	< 0.001
SFA (cm^2^)	186.65 ± 63.98	201.83 ± 61.52	201.81 ± 60.40	0.007
Smoking, n (%)	65 (26.21)	80 (32.00)	95 (38.31)	0.016
Drinking, n (%)	31 (12.55)	64 (25.81)	62 (24.80)	< 0.001
Hypertension, n (%)	89 (35.60)	91 (36.40)	102 (40.80)	0.434
Dyslipidemia, n (%)	101 (40.40)	106 (42.40)	111 (44.40)	0.664
Antihypertensive agents, n (%)	76 (30.40)	81 (32.40)	98 (39.20)	0.093
Lipid lowering agents, n (%)	55 (22.00)	68 (27.31)	74 (29.60)	0.141
Metformin therapy, n (%)	78 (34.21)	93 (40.09)	66 (31.13)	0.397
GLP-1 RA therapy, n (%)	3 (1.32)	3 (1.32)	3 (1.32)	0.703

Data are presented as mean ± standard deviation (SD), median (interquartile range), or n (%)

*Abbreviations*: FT3, free triiodothyronine; FT4, free thyroxine; DBP, diastolic blood pressure; SBP, systolic blood pressure; BMI, body mass index; FPG, fasting plasma glucose; HbA1c, glycated hemoglobin; LDL, low-density lipoprotein; HDL, high-density lipoprotein; TSH, thyrotropin; VFA, visceral fat area; SFA, subcutaneous fat area; GLP-1 RA, glucagon-like peptide-1 receptor agonist

## Correlations between TH sensitivity and VFA

Multivariate linear regression analysis revealed that each standard deviation (SD) increase in FT3 and FT3/FT4 was linked to increases of 5.24 cm^2^ [95% CI (2.03, 8.45)] and 4.65 cm^2^ [95% CI (1.63, 7.67)] in VFA, respectively, after full adjustment for confounding variables (Table 3). Furthermore, the middle and highest FT3/FT4 tertiles were significantly correlated with increases of 12.15 cm^2^ [95% CI (5.05, 19.25)] and 12.42 cm^2^ [95% CI (5.00, 19.84)] in VFA than the lowest tertile, respectively. However, a noteworthy correlation between VFA and TFQI, TT4RI, or TSHI was not observed.

**Table 3:** Associations of thyroid hormone sensitivity with VFA

	VFA, cm^2^				
	Model 1			Model 2	
	β (95% CI)	*P* value		β (95% CI)	*P* value
FT3 per SD	4.46 (1.66, 7.27)	0.002		5.24 (2.03, 8.45)	0.002
FT4 per SD	-1.59 (-4.34, 1.15)	0.255		-1.25 (-4.40, 1.90)	0.437
TSH per SD	1.80 (-0.94, 4.55)	0.198		-0.39 (-3.28, 2.49)	0.790
FT3/FT4 per SD	4.80 (2.05, 7.55)	< 0.001		4.65 (1.63, 7.67)	0.003
T 1	Reference			Reference	
T 2	13.48 (6.81, 20.15)	< 0.001		12.15 (5.05, 19.25)	< 0.001
T 3	12.36 (5.65, 19.06)	< 0.001		12.42 (5.00, 19.84)	0.001
*P* for trend	< 0.001			0.001	
TFQI per SD	2.33 (-0.47, 5.13)	0.103		1.22 (-1.86, 4.31)	0.438
T1	Reference			Reference	
T 2	2.79 (-3.93, 9.50)	0.416		2.08 (-5.12, 9.28)	0.571
T 3	5.63(-1.09, 12.34)	0.101		4.41 (-2.80, 11.62)	0.231
*P* for trend	0.101			0.229	
TT4RI per SD	0.91 (-1.83, 3.65)	0.514		-0.72 (-3.58, 2.15)	0.624
T 1	Reference			Reference	
T 2	2.49 (-4.21, 9.20)	0.466		2.31 (-4.91, 9.53)	0.531
T 3	3.43 (-3.26, 10.13)	0.315		-0.08 (-7.15, 6.99)	0.982
*P* for trend	0.315			0.953	
TSHI per SD	1.04 (-1.69, 3.78)	0.455		-0.75 (-3.81, 2.31)	0.631
T 1	Reference			Reference	
T 2	4.58 (-2.11, 11.27)	0.180		0.24 (-7.04, 7.51)	0.949
T 3	1.92 (-4.77, 8.61)	0.574		-1.63 (-8.72, 5.45)	0.651
*P* for trend	0.574			0.635	

Data are presented as standardized coefficients (β) and 95% confidence intervals (CI)

Model 1: Age and sex were adjusted

Model 2: Model 1 + adjustments for diabetes duration, FPG, uric acid, HbA1c, BMI, dyslipidemia, hypertension, drinking status, smoking status, antihypertensive agents, lipid lowering agents, metformin therapy and GLP-1 RA therapy

*Abbreviations*: VFA, visceral fat area; FT3, free triiodothyronine; SD, standard deviation; FT4, free thyroxine; TSH, thyrotropin; TFQI, thyroid feedback quantile-based index; TT4RI, thyrotrophic thyroxine resistance index; TSHI, TSH index

## Correlations of TH sensitivity with the prevalence of visceral obesity

Table 4 indicated that the middle and highest FT3/FT4 tertiles were correlated with a 134% [95% CI (1.24, 4.44)] and 98% [95% CI (1.04, 3.78)] higher prevalence of visceral obesity, respectively, compared to the lowest tertile, after full adjustment for confounding variables (*P* for trend = 0.032). TFQI was negatively associated with visceral obesity. The highest tertile of TFQI was linked to a 58% [95% CI (0.22, 0.83)] reduced prevalence of visceral obesity than the lowest tertile, after full adjustment for confounding variables (*P* for trend = 0.013).

**Table 4:** Odds ratios (OR) of thyroid hormone sensitivity indices to risk of visceral obesity (VFA ≥ 80 cm^2^)

	Visceral obesity				
	Model 1			Model 2	
	OR (95% CI)	*P* value		OR (95% CI)	*P* value
FT3 per SD	1.30 (1.08, 1.57)	0.005		1.64 (1.20, 2.23)	0.002
FT4 per SD	0.90 (0.75, 1.07)	0.222		1.16 (0.88, 1.53)	0.303
TSH per SD	1.09 (0.92, 1.30)	0.324		0.95 (0.74, 1.23)	0.721
FT3/FT4 per SD	1.34 (1.12, 1.61)	0.002		1.24 (0.94, 1.63)	0.132
T 1	Reference			Reference	
T 2	2.55 (1.65, 3.95)	< 0.001		2.34 (1.24, 4.44)	0.009
T 3	2.05 (1.34, 3.13)	< 0.001		1.98 (1.04, 3.78)	0.038
*P* for trend	< 0.001			0.032	
TFQI per SD	1.00 (0.83, 1.19)	0.990		0.75 (0.57, 1.00)	0.046
T1	Reference			Reference	
T 2	0.92 (0.60, 1.42)	0.711		0.52 (0.27, 1.01)	0.052
T 3	0.84 (0.55, 1.30)	0.439		0.42 (0.22, 0.83)	0.012
*P* for trend	0.438			0.013	
TT4RI per SD	1.04 (0.87, 1.23)	0.688		0.98 (0.76, 1.27)	0.898
T 1	Reference			Reference	
T 2	1.21 (0.79, 1.86)	0.375		1.62 (0.85, 3.10)	0.145
T 3	1.18 (0.77, 1.80)	0.444		1.13 (0.61, 2.10)	0.705
*P* for trend	0.442			0.738	
TSHI per SD	1.05 (0.88, 1.24)	0.611		1.07 (0.82, 1.42)	0.608
T 1	Reference			Reference	
T 2	1.14 (0.74, 1.76)	0.546		1.49 (0.78, 2.85)	0.229
T 3	0.98 (0.65, 1.50)	0.938		1.03 (0.55, 1.94)	0.928
*P* for trend	0.937			0.984	

The evaluation of the OR and 95% confidence interval (CI) was conducted using multivariable logistic regression model

Model 1: Age and sex were adjusted

Model 2: Model 1 + adjustments for diabetes duration, FPG, uric acid, HbA1c, BMI, dyslipidemia, hypertension, drinking status, smoking status, antihypertensive agents, lipid lowering agents, metformin therapy and GLP-1 RA therapy

*Abbreviations*: VFA, visceral fat area; FT3, free triiodothyronine; SD, standard deviation; FT4, free thyroxine; TSH, thyrotropin; TFQI, thyroid feedback quantile-based index; TT4RI, thyrotrophic thyroxine resistance index; TSHI, TSH index

### Stratified analysis for correlations of FT3/FT4 and TFQI with visceral obesity

**Fig. 1:**
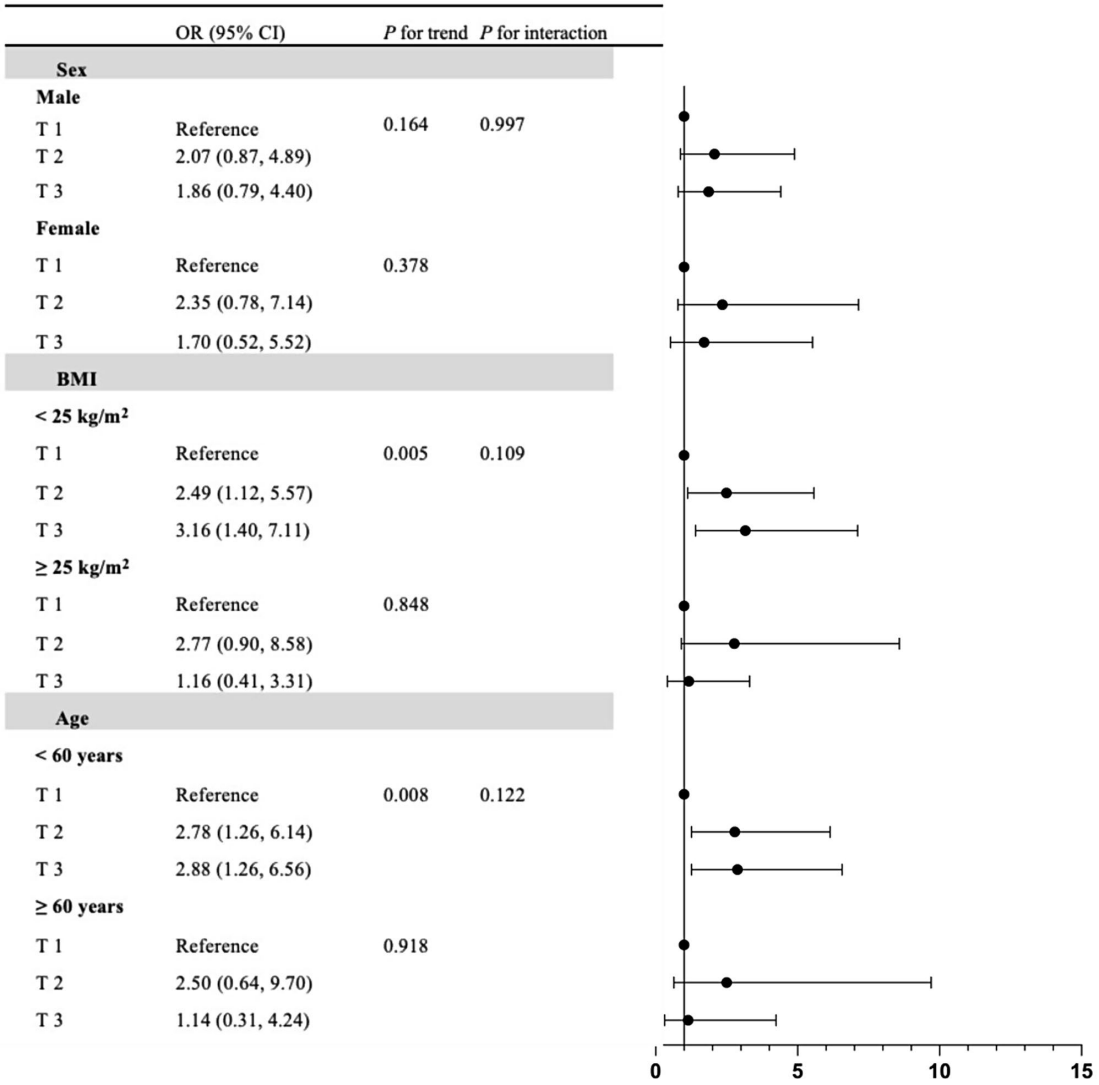
Stratified analysis of the correlation between FT3/FT4 and visceral obesity (VFA ≥ 80 cm^2^). Adjusted for age, sex, FPG, uric acid, HbA1c, BMI, diabetes duration, dyslipidemia, hypertension, drinking status, smoking status, antihypertensive agents, lipid lowering agents, metformin therapy and GLP-1 RA therapy, if not be stratified. *Abbreviations*: FT3, free triiodothyronine; FT4, free thyroxine; VFA, visceral fat area; OR, odds ratio; CI, confidence interval; BMI, body mass index

**Fig. 2:**
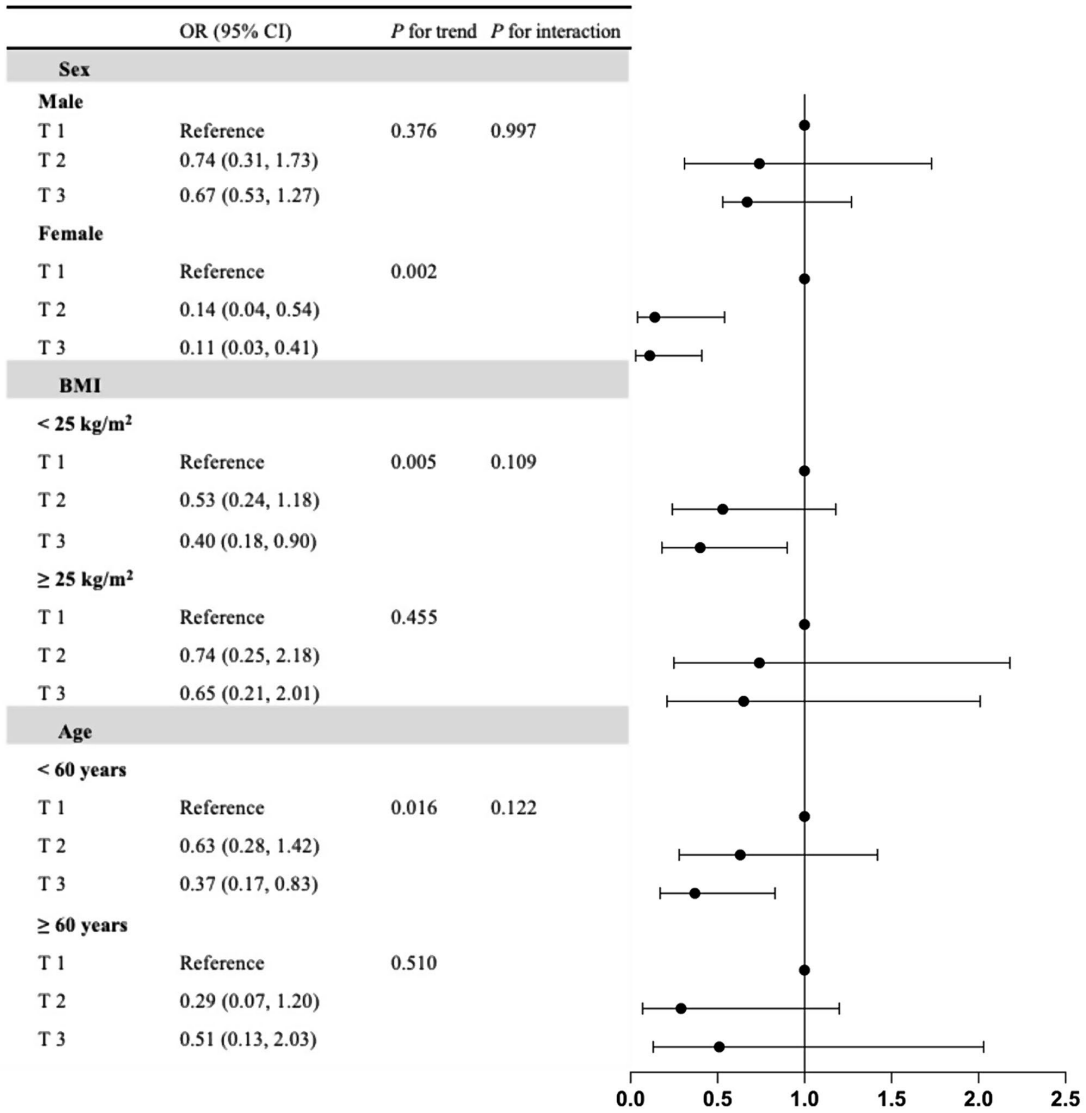
Stratified analysis of the correlation between TFQI and visceral obesity (VFA ≥ 80 cm^2^). Adjusted for age, sex, FPG, uric acid, HbA1c, BMI, diabetes duration, dyslipidemia, hypertension, drinking status, smoking status, antihypertensive agents, lipid lowering agents, metformin therapy and GLP-1 RA therapy, if not be stratified. *Abbreviations*: FT3, free triiodothyronine; FT4, free thyroxine; VFA, visceral fat area; OR, odds ratio; CI, confidence interval; BMI, body mass index

Furthermore, stratified analyses were conducted to examine the relationship of visceral obesity with TFQI and FT3/FT4 according to the potential modifiers, including age, BMI and sex. As illustrated in Figs. 1 and 2, the middle and highest FT3/FT4 tertiles were significantly correlated with higher prevalence of visceral obesity only among subjects with BMI < 25 kg/m^2^ and those younger than 60 years, compared to the lowest tertile. The highest TFQI tertile was correlated with reduced prevalence of visceral obesity among females and subjects with BMI < 25 kg/m^2^ and those younger than 60 years, compared to the lowest tertile, after full adjustment for confounding variables (all *P* < 0.05). No modification effect was detected.

### The nonlinear relationship between VFA and FT3/FT4

**Fig. 3:**
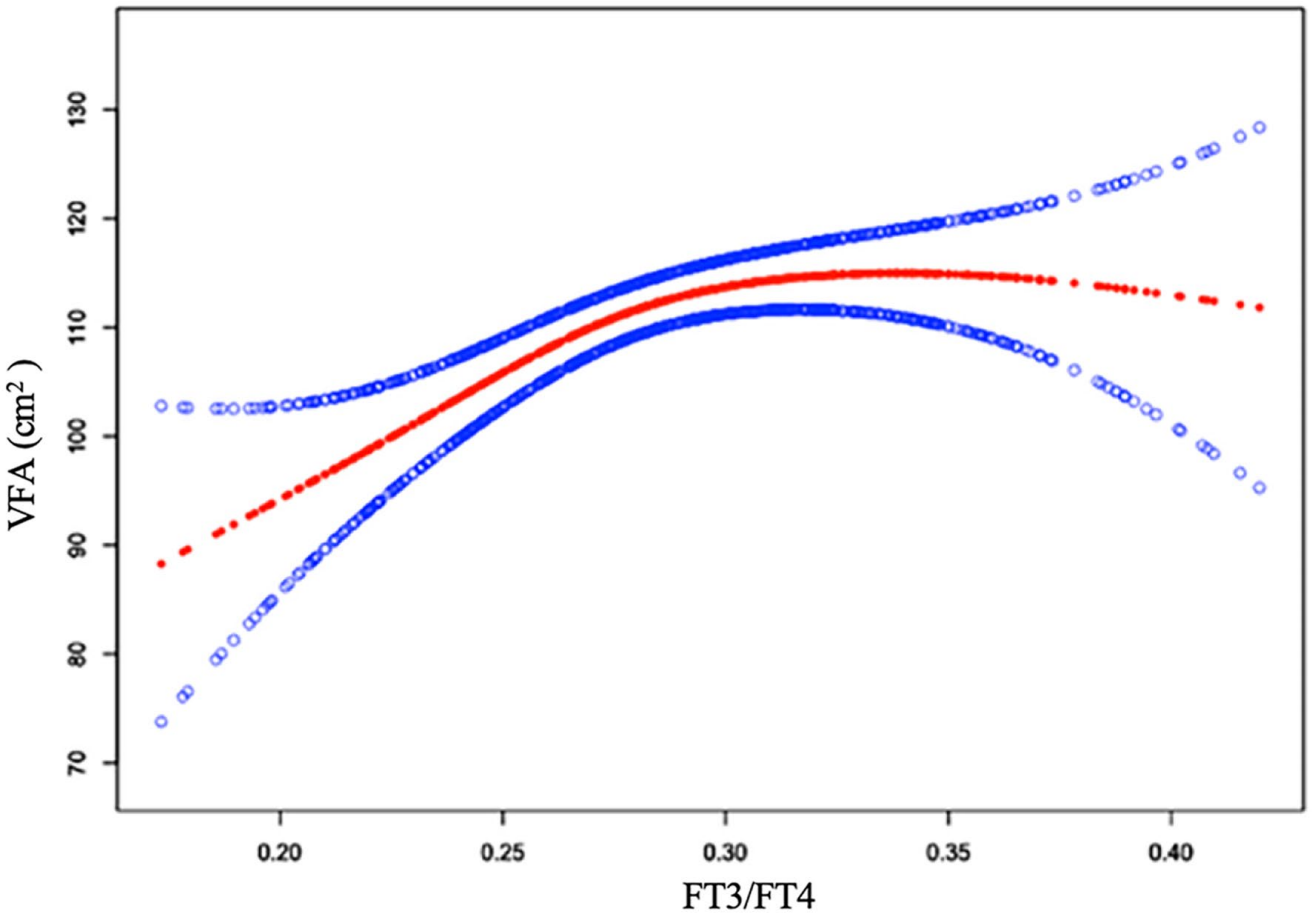
Smooth curve fitting model for nonlinear relationship of FT3/FT4 with VFA. The fitted curve is depicted by the red line, while the confidence interval is shown by blue lines. Adjusted for age, sex, FPG, uric acid, HbA1c, BMI, diabetes duration, dyslipidemia, hypertension, drinking status, smoking status, antihypertensive agents, lipid lowering agents, metformin therapy and GLP-1 RA therapy. *Abbreviations*: FT3, free triiodothyronine; FT4, free thyroxine; VFA, visceral fat area

Based on the findings from the stratified analysis, we hypothesized that a nonlinear correlation exists between FT3/FT4 and VFA. Employing a model with smooth curve fitting, a positive, nonlinear correlation of FT3/FT4 with VFA was observed after adjusting the confounding variables (Fig. 3). The inflection point for FT3/FT4 was identified at 0.28 (log-likelihood ratio test *P* = 0.03), calculated by binary linear regression model and recursive algorithm.

## Discussion

This study establishes a positive relationship between FT3/FT4 and VFA, as well as visceral obesity, as assessed by VFA ≥ 80 cm^2^, after adjusting for potential confounders in euthyroid patients with T2D. Meanwhile, TFQI was negatively associated with visceral obesity. These relationships are particularly pronounced among participants who are neither overweight nor obese and those younger than 60 years, suggesting that VFA and visceral obesity are positively associated with both peripheral and central TH sensitivity.

Adipose tissue is instrumental in regulating systemic insulin sensitivity, energy expenditure, and body weight [3, 32]. In instances where subcutaneous adipose tissue cannot undergo hyperplasia of preadipocytes to adapt to overfeeding, the deposition of visceral and ectopic fat ensues [5]. This excessive visceral and ectopic fat accumulation may contribute to a spectrum of metabolic abnormalities and diseases [2, 3, 5, 33]. THs regulate systemic energy homeostasis through white adipose tissue [34]. Furthermore, the application of synthetic TH has been observed to improve lipid metabolism, leading to a decrease in body weight. Several TRβ-specific agonists have shown promising therapeutic effects in animal models of nonalcoholic fatty liver disease [35]. The FDA has granted approval for the use of resmetirom for treating noncirrhotic nonalcoholic steatohepatitis in adults with moderate to advanced liver fibrosis, in conjunction with dietary and exercise interventions [36]. Recent research has revealed that adipose-targeted T3 therapy can ameliorate obesity-related metabolic disorders and atherosclerosis with minimal adverse effects [37]. Thus, understanding the relationship between circulating TH levels, TH sensitivity, and visceral fat accumulation, as well as the precise TH status in visceral adipose tissue in individuals with T2D, is crucial. This study offers novel insights for forthcoming research on TH and adipose tissue in the context of metabolic disease.

Previous investigations into the associations between FT3, FT3/FT4, and VFA have produced varied outcomes. Lv et al. identified a negative relationship between VFA and FT3/FT4 in a Chinese euthyroid cohort post adjustment for sex, age, and BMI [21], whereas an earlier study found no significant relationship between VFA and either FT3 or FT3/FT4 across genders [23]. This research, however, confirms a marked positive association between FT3/FT4 and VFA as well as visceral obesity in euthyroid patients with T2D after accounting for confounders, aligning with certain prior findings [22]. Additionally, in euthyroid subjects, there have been positive correlations noted between FT3, FT3/FT4, and metabolic markers such as the triglyceride-glucose index [38], WC, BMI, TG, FPG [39], and nonalcoholic fatty liver disease [40, 41].

Deiodinases allow for the tissue-specific regulation of intracellular TH levels, independent of plasma TH concentrations [11, 13]. Deiodinases (DIO) 1 or DIO2 catalyzes the conversion of T4 to T3 [11, 13]. Bradley et al. observed an upregulation of DIO2 in both visceral and subcutaneous adipocytes in obese subjects than lean counterparts, with a significant association of higher DIO2 levels with reduced mitochondrial function and fatty acid oxidation, irrespective of diabetes status [42]. Moreover, high carbohydrate diets have been linked with substantially elevated serum T3 levels in comparison to very low carbohydrate diets [43], suggesting a critical physiological adaptation during nutrient surplus.

Interestingly, stratified analyses revealed a positive correlation of FT3/FT4 with both VFA and visceral obesity, exclusively in participants who are neither overweight nor obese. Danforth et al. demonstrated that short-term overfeeding (3 weeks) led to increased T3 concentrations, with no changes observed following long-term (3 months) fat overfeeding [44]. Additionally, TH receptor and DIO2 expression in adipose tissue from morbidly obese individuals was significantly reduced than in those of normal weight [45]. These observations imply a potential impairment in the physiological adaptation capacity among individuals with morbid obesity and chronic overnutrition.

It is particularly noteworthy that the association between FT3/FT4 and VFA as well as visceral obesity is observed predominantly in individuals under the age of 60. The mechanisms underlying this specific correlation remain unclear. Notably, the activity of deiodinases, crucial for TH metabolism, changes throughout an individual’s life, reflecting the differing requirements of various organs and the aging process [46, 47].

Additionally, Lv and his colleagues identified a positive correlation between PTFQI, TFQI, TSHI and VFA in a euthyroid Chinese population [21]. In contrast, elevated PTFQI, TFQI and TSHI levels were associated with a reduced prevalence of obesity in individuals with subclinical hypothyroidism [19]. Liu et al. reported that individuals with metabolically healthy obesity demonstrated greater central TH sensitivity compared with those with metabolically healthy non-obesity. While TFQI and TSHI showed a positive correlation with metabolic abnormality in subjects with obesity [48]. This study demonstrated that higher level of TFQI was linked to decreased prevalence of visceral obesity in euthyroid individuals with T2D. These associations were particularly pronounced in female participants and those who are neither overweight nor obese. Therefore, it is reasonable to hypothesize that an early compensatory increase in TH secretion might occur in individuals with abdominal obesity. Alternatively, there may exist an underlying mechanism that facilitates heightened central TH sensitivity in women or individuals with a BMI < 25 kg/m^2^ among abdominally obese population. However, a noteworthy correlation between visceral obesity and TT4RI or TSHI was not observed. TFQI is calculated by a cumulative distribution function of TSH and FT4, so it is not prone to extreme values compared to TT4RI and TSHI, even in the presence of abnormal thyroid function [15]. This may explain why a statistically significant association was observed between TFQI and abdominal obesity, while no such correlation was found between TT4RI or TSHI and abdominal obesity.

### Study strengths and limitations

A major strength is the meticulous adjustment for potential confounders, particularly pharmacological interventions that may influence VFA, such as lipid-lowering drugs, metformin, and GLP-1RA. Additionally, the stratified analyses unveiled intriguing outcomes, including a positive, non-linear relationship between FT3/FT4 and VFA, explored through a model employing smooth curve fitting. However, the study is not without its limitations. Firstly, a causal relationship between TH sensitivity indices and VFA or adipose tissue cannot be confirmed due to the cross-sectional design. Secondly, since this cohort consists solely of Chinese participants, the applicability of these findings to other ethnic and racial groups is uncertain and warrants further exploration. Thirdly, although FT3/FT4 can reflect thyroid deiodination, it may not fully reflect the local action of TH in adipose tissue, this warrants more research to explore the relationship between adipose tissue and TH in obese states.

## Conclusions

This analysis revealed that increased TH sensitivity is significantly linked to both visceral obesity and elevated VFA in euthyroid individuals with T2D, particularly among those younger than 60 years and individuals who are neither overweight nor obese. These insights offer substantial evidence towards understanding the complex association between visceral adipose tissue and TH sensitivity more clearly, while also lay the groundwork for the development of potential weight loss drugs aimed at adipose tissue, such as TH analogs.

## Electronic supplementary material

Below is the link to the electronic supplementary material.

Supplementary Material 1

Supplementary Material 2

Supplementary Material 3

## Data Availability

No datasets were generated or analysed during the current study.

## Abbreviations

T2D: Type 2 diabetes
TT4RI: Thyrotrophic thyroxine resistance index
TSHI: Thyrotropin index
TFQI: Thyroid feedback quantile-based index
VFA: Visceral fat area
TSH: Thyrotropin
BMI: Body mass index
SFA: Subcutaneous fat area
HDL-C: High-density lipoprotein cholesterol
LDL-C: Low-density lipoprotein cholesterol
TG: Triglyceride
TC: Total cholesterol
DBP: HDiastolic blood pressureC
SBP: Systolic blood pressure
HbA1c: Glycated hemoglobin
FPG: Fasting plasma glucose
GLP-1 RA: Glucagon-like peptide-1 receptor agonist
WC: Waist circumference
SD: Standard deviation
DIO: Deiodinases
