# Supplementary material for: Increased thyroid hormone sensitivity is correlated with visceral obesity in patients with type 2 diabetes
Source: Lipids Health Dis. 2024 Oct 16;23:337. doi: 10.1186/s12944-024-02320-9 (PMC11481250; doi:10.1186/s12944-024-02320-9)
Supplement: Supplementary file 1 — Supplementary Material 1 [file 12944_2024_2320_MOESM1_ESM.pdf]

瑞金医院涉及人体科研项目伦理委员会补充审批件
Approval Form Supplement of CTEC ,Ruijin Hospital

（2017）临伦审第（42）号-3

原伦审批件号 IEC approval	（2017）临伦审第（42）号
临床试验名称 Project	代谢性疾病的血清学、代谢组学和基因组学研究
经费来源 Support	“精准医学研究”重点专项—代谢性疾病专病队列研究-2016YFC0901200
项目负责人 PI	宁光

Document	Version	Date
修正申请	NA	2021-12-27
研究方案	NA	2021-12-27
知情同意书	2.0	2021-12-27

伦理委员会已接收上述文件，经审核，符合伦理规范，同意进行临床研究。

瑞金医院伦理委员会（签章）：
Ruijin Hospital Ethics Committee（stamp）
主任委员（签章）：
Signature:

日期：
Date : 2022.3.9

地址：上海市瑞金二路 197 号
邮编：200025
电话：0086-021-54661789

No.197 Ruijin Er Road
Shanghai, P.R China,200025
Tel: 0086-021-54661789
