# Supplementary material for: Increased thyroid hormone sensitivity is correlated with visceral obesity in patients with type 2 diabetes
Source: Lipids Health Dis. 2024 Oct 16;23:337. doi: 10.1186/s12944-024-02320-9 (PMC11481250; doi:10.1186/s12944-024-02320-9)
Supplement: Supplementary file 3 — Supplementary Material 3 [file 12944_2024_2320_MOESM3_ESM.pdf]

EDITSPRINGS

EDITORIAL CERTIFICATE

This is to certify that the manuscript detailed below was edited by one or more of our highly qualified, native English speakers at EditSprings, to assure compliance with Anglophone academic standards in terms of style, punctuation, grammar, and spelling.

Manuscript title:

Increased thyroid hormone sensitivity is correlated with visceral obesity in patients with type 2 diabetes

Authors:

Lu Yu, Yujia Liu, Yingxuan Wang, Gang Wang, Xianchao Xiao, Huan Wang, Hanyu Wang, Hui Sun, and Guixia Wang

Date Issued:

Sep 30 2024

Certificate Number:

ES-202409271204539337

This certificate can be verified on https://www.editsprings.com/QueryCertificate.html . EditSprings hereby certifies that neither content nor the author's intentions were altered in any way during the editing process. Documents in receipt of this certification should be ready for publication as far as style and language are concerned, provided that the author(s) accepted our suggestions and changes (which remains their right and responsibility).

EditSprings offers a wide range of editing, translation, for researchers and publishers across the world. Our highly skilled editors are all established academics based in Anglophone Higher Education institutions across the world (U.K., U.S.A., Canada, Australia, and elsewhere), are experts in their respective fields, and are qualified to edit research papers authored by non-Anglophone scholars.
